# Dynamic Interplay between Pericytes and Endothelial Cells during Sprouting Angiogenesis

**DOI:** 10.3390/cells8091109

**Published:** 2019-09-19

**Authors:** Giulia Chiaverina, Laura di Blasio, Valentina Monica, Massimo Accardo, Miriam Palmiero, Barbara Peracino, Marianela Vara-Messler, Alberto Puliafito, Luca Primo

**Affiliations:** 1 Candiolo Cancer Institute-FPO, IRCCS, Str. Prov. 142, km 3.95, 10060 Candiolo, Italy; giulia.chiaverina@ircc.it (G.C.); laura.diblasio@ircc.it (L.d.B.); valentina.monica@unito.it (V.M.); massimo.accardo@ircc.it (M.A.); miriam.palmiero@ircc.it (M.P.); marianela.varamessler@ircc.it (M.V.-M.); alberto.puliafito@ircc.it (A.P.); 2 Department of Oncology, University of Turin, 10060 Candiolo, Italy; 3 Department of Clinical and Biological Sciences, University of Turin, San Luigi Hospital, 10043 Orbassano, Italy; barbara.peracino@unito.it

**Keywords:** pericytes, endothelial cells, aortic ring assay, sprouting angiogenesis, NG2, cancer

## Abstract

Vascular physiology relies on the concerted dynamics of several cell types, including pericytes, endothelial, and vascular smooth muscle cells. The interactions between such cell types are inherently dynamic and are not easily described with static, fixed, experimental approaches. Pericytes are mural cells that support vascular development, remodeling, and homeostasis, and are involved in a number of pathological situations including cancer. The dynamic interplay between pericytes and endothelial cells is at the basis of vascular physiology and few experimental tools exist to properly describe and study it. Here we employ a previously developed ex vivo murine aortic explant to study the formation of new blood capillary-like structures close to physiological situation. We develop several mouse models to culture, identify, characterize, and follow simultaneously single endothelial cells and pericytes during angiogenesis. We employ microscopy and image analysis to dissect the interactions between cell types and the process of cellular recruitment on the newly forming vessel. We find that pericytes are recruited on the developing sprout by proliferation, migrate independently from endothelial cells, and can proliferate on the growing capillary. Our results help elucidating several relevant mechanisms of interactions between endothelial cells and pericytes.

## 1. Introduction

Pericytes are perivascular cells that can be distinguished from vascular smooth muscle cells (VSMC) by their specific morphology, molecular markers expression, and tissue distribution [1,2]. Differently from VSMC, which cover large-diameter vessels like arteries and veins, pericytes are mural cells mainly localized in the microvasculature where they cover precapillary arterioles, capillaries, and postcapillary venules [2]. Pericytes identification with molecular markers is not trivial because of the lack of specific markers suitable to label all types of pericytes, independently from tissue of origin and function [2,3,4,5], such as those modulating blood flow or originating from the endothelial brain barrier [2,6,7].

Pericytes also play an important functional role in angiogenesis, the formation of new blood vessels from pre-existing ones. This dynamic biological process starts with the sprouting of endothelial cells (ECs) from pre-existing vessels, followed by the elongation of a functional tube and the re-introduction of perivascular cells [2,8,9]. Pericytes, embedded into the vascular basement membrane (vBM) shared with EC [1,2], detach, in response to Angiopoietin-2, from the vessel wall by proteolytic degradation of vBM, then by promoting EC migration in the surrounding extracellular matrix [10].

The Platelet-derived growth factor subunit B (PDGF-B) released by EC is the main soluble signal involved in the pericytes recruitment and vascular stabilization [11]. The lack of PDGF-B and the corresponding absence of pericytes has been correlated to endothelial hyperplasia, increased capillary diameter, and abnormal ECs shape [12]. Pericyte deficiency results in vBM defects or reduction, as ECs-pericytes interactions enhance the deposition of fibronectin, nidogen-1, laminins, and other vBM components [13,14]. Pericytes are also necessary to support the endothelial layer maturation by the release of several paracrine factors suppressing ECs proliferation and leading to a quiescent vessel, notably transforming growth factor beta (TGFβ) and angiopoietin-1 [15].

Numerous biomarkers have been identified to specifically identify pericytes from other mesenchymal cells. One of the most used marker is neuron-glial 2 chondroitin sulfate proteoglycan (NG2) [4,16]. NG2 has often been considered a suitable pericyte marker for studies of arterial biology and vascular growth, as NG2 expression is restricted to arteriolar and capillary perivascular cells during vasculogenesis and angiogenesis [16]. Although the functional role of the proteoglycan in vascular development is yet to be fully understood, several properties of NG2 are suggestive of its function in mural cell biology. For example, NG2 is a putative extracellular matrix ligand of collagen type VI, a BM component in some types of vasculature, suggesting a role in pericyte–vBM interactions required for vascular development [4].

Other proteoglycans are actively involved in the angiogenic process [17]. Perlecan, the major heparan sulfate proteoglycan of BMs, plays active roles in both tumor invasion and endothelial cell migration in angiogenesis, supporting integrin-mediated adhesion and modulation of VEGFA-VEGFR2 signaling [18,19]. Likewise, agrin participates in tumor vascularization, regulating EC invasion and migration via integrin β1-Lrp4 axis and promoting VEGFR2 stability and activation, thereby representing a hypothetical target to reduce vascularization within tumors [20].

Another classic marker used for pericytes identification is alpha-smooth muscle actin (αSMA), whose expression is related to the pericytes regulatory function of capillaries blood flow, modulating vasoconstriction of arteriole and capillaries via endothelin-1 signaling [2]. Moreover, αSMA is often absent in quiescent pericytes in normal tissues, but readily detected in pathological conditions such as tumor angiogenesis, tissue fibrosis, and inflammation [4,16].

A third commonly adopted pericyte marker is platelet-derived growth factor receptor-beta (PDGFRβ), which is functionally involved in pericytes recruitment during angiogenesis and it is expressed broadly on developing vSMCs. PDGFRβ also plays a role in the proliferation and differentiation of aortic and venous vSMCs [1,21,22]. Note that many of the markers commonly applied to identify pericytes are neither specific nor stable in their expression [1,2].

Although the presence of pericytes in the vasculature has been widely documented in the past, a renewed effort is currently dedicated to study pericytes lineage, function, and motility, especially in association with ECs [23,24].

Given the increasing attention paid to these cells and their functional relevance in physiological and pathological angiogenesis, it is relevant to clarify what drives pericyte vascular coverage. Little is known about where they originate from and how they behave once they reach the newly formed vessel, whether they are static or able to move and undergo cell division. The role of pericytes is typically studied on static fixed tissues and a truly dynamic characterization is still far from being achieved.

Frequently, human pericytes isolated on the basis of different expression markers and cultured on plastic surface lose their morphological features, and eventually dedifferentiate and lose their specific markers [25]. Furthermore, from a biological viewpoint, pericytes assume a specific relevance and function only with respect to their multiple interactions with the surrounding microvasculature components, like ECs and vBM. In addition, the biological model systems suitable for the study of multicellular angiogenic process are few and often not amenable to culture needs, making the study of the whole EC–pericyte system very complicated and hard to approach experimentally.

To overcome these limitations, we took advantage of the ex vivo mouse aortic ring (mAR) model to study pericyte dynamics in sprouting angiogenesis [26]. This assay is characterized by the VEGF-induced sprouting of capillary-like structures from cultured murine aortic sections. Developing microvessels undergo many key features of angiogenesis over a timescale similar to that observed in vivo [26,27,28,29].

We exploited transgenic mice that stably express the dsRed fluorescent protein under the NG2 promoter, thereby labeling pericytes [30]. The mAR assay was then exploited to monitor pericytes during sprouting angiogenesis. Thanks to NG2-dsRed mice crossed with LifeAct-EGFP [31] or H2B-EGFP-transgenic mice [32], we generated a model amenable to live microscopy studies of pericytes dynamics in sprouting angiogenesis. Our results follow.

## 2. Materials and Methods

### 2.1. Animals

NG2-dsRed mice (stock 008241) were purchased from The Jackson Laboratory. LifeAct–EGFP mice were generated previously [31], and provided by R. Wedlich-Söldner (Max-Planck Institute of Biochemistry, Martinsried, Germany) and L. M. Machesky (Beatson Institute for Cancer Research, Glasgow, UK). H2B-EGFP mice (stock 006069) were purchased from The Jackson Laboratory.

Approximately 30 mice were used to perform the described experiments. Mice were housed under the approval and the institutional guidelines governing the care of laboratory mice of the Italian Ministry of Health, under authorization number 1073/2015-pr and in compliance with the international laws and policies.

### 2.2. Mouse Aortic Ring Angiogenesis Assay

The mouse aortic ring (mAR) assay was performed as previously described [26,29,33] with the following modifications. After explant, 12 mARs per aorta were incubated O/N in serum-free medium. Aortic explants were then kept in place on glass-bottom dishes (WillCo Wells, Amsterdam, Netherlands) with a drop of 20 μL of type-I collagen gel (from rat tail, Roche, Manheim, Germany) and was covered with Endothelial Basal Medium-2 (EBM-2, CC-3156, Lonza, Basel, Switzerland) with FBS, hFGF-B, VEGF, GA-1000, and Heparin from EGMTM-2 SingleQuotsTM Supplements (CC-4176, Lonza).

### 2.3. Immunostaining

Whole-mount mARs or mA-sheets were equilibrated in PBS, PAF-fixed, and permeabilized with PBS 0.5% Triton X-100 for 3 h. Blocking incubation was performed for 1 h in IF buffer (PBS 0.2% Triton X-100, 0.05% Tween-20, 10% donkey serum). Primary antibodies—rabbit anti-laminin (1:100, AB19012, Merck, Darmstadt, Germany), rat anti-PECAM-1 (MEC 13.3, 1:100, sc-18916, Santa Cruz, Santa Cruz, CA, USA), rabbit anti-NG2 Chondroitin Sulfate Proteoglycan (1:100, AB5320, Millipore, Darmstadt, Germany), rabbit anti-alpha smooth muscle Actin (1:100, ab5694, Abcam, Cambridge, UK), goat anti-mVE-cadherin (1:80, AF1002, R&D Systems, Minneapolis, MN, USA), and rabbit anti-PDGF Receptor β (28E1, 1:100, 3169S, Cell Signaling Technology, Danvers, MA, USA)—were diluted in IF Buffer and incubated overnight at 4 ${}^{\circ }$C in a humidified chamber. Specimens were washed with PBS and incubated for 1 h at room temperature with secondary antibodies (Invitrogen, Carlsbad, CA, USA) diluted 1:200 in PBS and counterstained with 4${}^{\prime }$,6-diamidino-2-phenylindole (DAPI). Specimens were analyzed using a confocal laser-scanning microscope (TCS SPE AOBS; Leica Microsystems, Wetzlar, Germany).

### 2.4. Time-Lapse Microscopy of mAR

mARs were embedded in type-I collagen gel, cultured as described previously, and kept at 37 ${}^{\circ }$C in a 5% CO${}_{2}$ humidified atmosphere on glass-bottom dishes. Time-lapse live imaging was started on the 5th or 6th day of the assay. mARs were imaged with a 20×/0.75 dry objective (Leica Microsystems) with an inverted motorized widefield microscope (AF6000 LX Leica Microsystems) in bright field or epifluorescence. Confocal images were acquired with an inverted Leica SP8 with a dry 20× objective. Z stacks were acquired every 1 to 5 μm z-step size, and every 30 min, over 36 h.

### 2.5. Image Analysis

The image and data analysis was performed by means of custom written scripts in Matlab (Mathworks, Natick, MA, USA). Cell tracking was implemented by marking the position of cells in each stack manually and then identifying cells automatically and tracking by minimum distance criterion. Velocity was calculated by finite differences starting from the position of cells.

## 3. Results

### 3.1. NG2-dsRed Mouse Aortic Ring Sprouting Assay Is a Valid Biological Model to Study Pericyte Dynamics

The mouse aortic ring (mAR) sprouting assay has been widely used in the past to recapitulate the intrinsic complexity of the process of vessel formation [26,28]. When embedded in collagen type I and cultured in liquid media (see details in Section 2), mARs generate sprouts of EC that grow and invade the ECM, forming capillary-like structures. This process can then be observed with a variety of microscopy techniques [26]. To verify the validity of this model so as to study pericytes in sprouting angiogenesis, we first characterized perivascular cells found along mAR sprouts.

**Figure 1:**
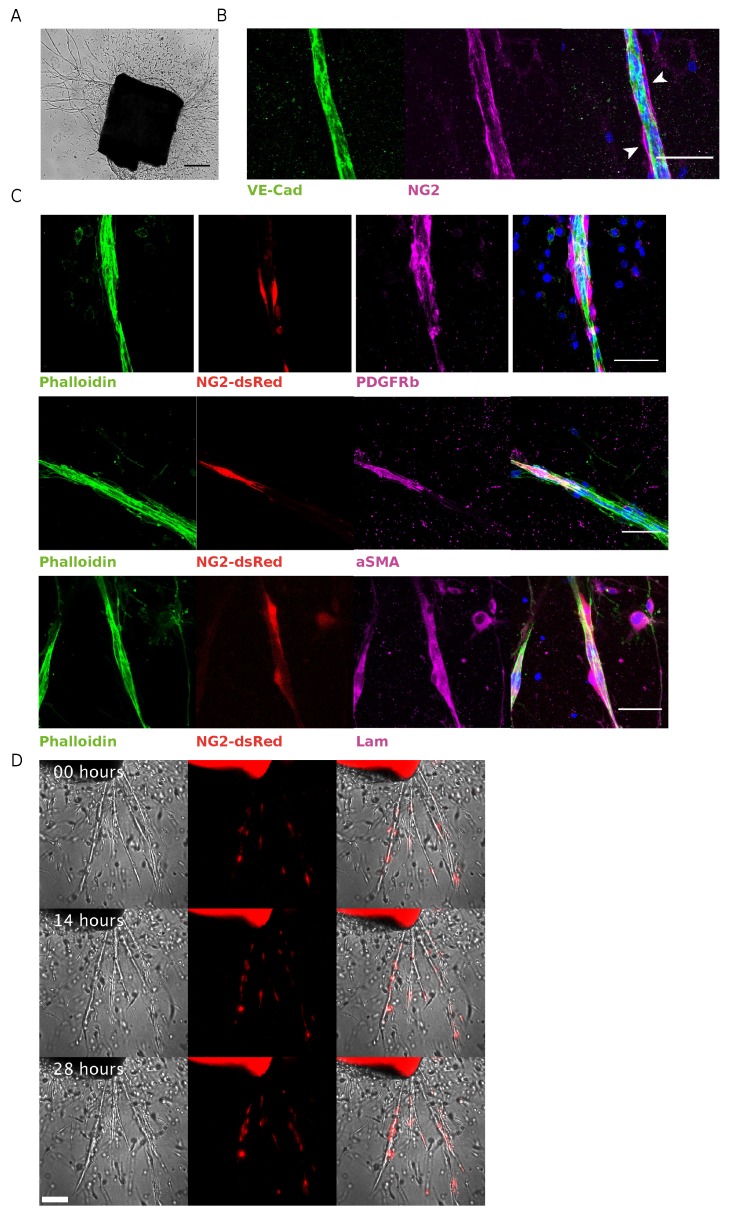
Characterization of NG2-dsRed mAR. (**A**) Representative bright-field image of mouse aortic ring cultured for one week, showing microvessel outgrowth (scale bar represents 200 μm). (**B**) Whole-mount immunofluorescence staining of a mAR fixed after 6 days of culture. The mAR was stained for the endothelial marker VE-cadherin (green) and the pericyte marker NG2 (magenta) (Scale bar: 50 μm). (**C**) mARs obtained from NG2-dsRed mouse model were cultured for 6 days then fixed to perform a whole-mount immunofluorescence staining. mARs were stained for classical pericyte markers, like PDGFRβ (magenta, top row) and αSMA (magenta, middle row), to verify the colocalization with dsRed signal (red). mARs were also stained for laminin (magenta, bottom row), one of the main components of vBM. Phalloidin staining was performed to identify microvessel outgrowths (green) (Scale bar: 50 μm). (**D**) Snapshots of time-lapse microscopy of angiogenic outgrowths from NG2-dsRed mAR.

Aortic explants usually start sprouting after 3–4 days post VEGF stimulation (see Figure 1A). We assessed the presence of pericytes in capillary-like structures after 7–8 days of culture by whole-mount immunofluorescence. The presence of pericytes was confirmed by identifying NG2 positive cells surrounded by VE-cadherin-positive regions corresponding to the endothelial layer as shown in Figure 1B.

To better exploit the mAR model in the study of pericytes dynamics, we took advantage of mice expressing dsRed under NG2 promoter (NG2-dsRed), thereby generating a biological tool to easily identify and study NG2-positive pericytes and their interactions with the endothelial layer during sprouting angiogenesis.

We verified the presence of dsRed positive cells in capillary-like structures sprouted from mAR, and the expression of different pericytes markers in those cells. On endothelial sprouts we found dsRed expressing cells which were also positive for PDGFRβ and αSMA (see Figure 1C top and middle rows), thus confirming that fluorescent cells correspond to pericytes in our model. Moreover, few perivascular cells are PDGFRβ-positive but dsRed negative, indicating a degree of pericytes heterogeneity even in this model.

Pericytes are usually embedded in the vBM to whose deposition they contribute during tube morphogenesis [14]. The vBM also encompasses the majority of the pericyte–endothelial interface where important interactions between the two cell types regulate vascular development, stabilization, remodeling, and physiology [34]. We analyzed the distribution of laminin, one of the main components of vBM, and identified a thin laminin-positive layer surrounding the NG2-positive cells (Figure 1C bottom row).

Thus, we further confirmed the validity of the NG2-dsRed mAR model to identify and dynamically monitor pericytes in their physiological microenvironment, with the possibility to further investigate the interaction between pericytes and the vBM.

We then made use of suitable microscopy techniques that would allow to follow pericytes dynamics. We performed live observations of vessel formation with mAR NG2-dsRed by time-lapse microscopy recognizing NG2-dsRed-positive pericytes moving over the aortic sprouts during vessel elongation (Figure 1D and Supplementary Movie S1). This experimental approach allows the detailed observation of pivotal events of sprouting angiogenesis and pericyte coverage, which have an in vivo counterpart.

The average number of pericytes per sprout is 2.2 (N=55 sprouts), with an average linear density (at the end of the assay) of 6.5 cells/mm (average sprout length: 336μm).

### 3.2. EC–Pericyte Interactions Can Be Studied at Single Cell Resolution by Means of LifeAct-EGFP/H2B-EGFP NG2-dsRed mAR

**Figure 2:**
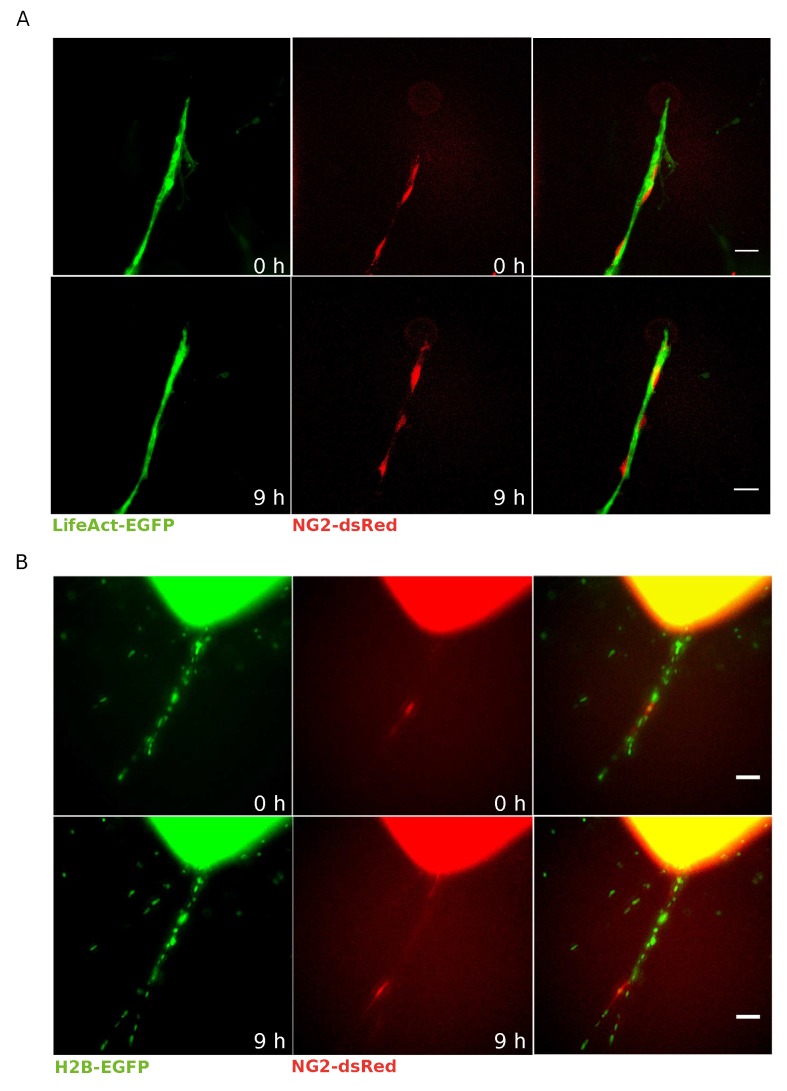
LifeAct-EGFP or H2B-EGFP NG2-dsRed mAR models for pericyte–EC interactions. (**A**) Time-lapse microscopy was performed on LifeAct-EGFP NG2-dsRed mARs cultured for 5 days then imaged for 72 h. This mouse model allows to clearly identify pericytes thanks to dsRed signal (red) running over the endothelial layer labeled by LifeAct-EGFP (green) (scale bar: 50 μm). (**B**) Time-lapse microscopy of H2B-EGFP NG2-dsRed mARs cultured for 5 days then imaged for 72 h. Thanks to this model we could identify pericytes (red) as well as track all individual cells thanks to histone H2B-EGFP (green) (scale bar: 50 μm).

EC–pericyte interactions in the blood vessel wall are crucial in the regulation of vascular development, stabilization, remodeling, and function [2,12,34,35]. To obtain a model suitable to describe simultaneously ECs and pericytes, we crossed mice that stably express LifeAct-EGFP [31] with NG2-dsRed mice (Figure 2A). Thanks to this model, where the LifeAct-EGFP signal is particularly bright on ECs and therefore is very effective in labeling ECs [33,36], we were able to identify and selectively study NG2-positive pericytes running over the endothelial layer during the sprout formation of a mAR (Figure 2A and Supplementary Movie S2). As a side note, live microscopy of LifeAct-EGFP NG2-dsRed mAR allows investigating, in particular, the protrusive activity of single ECs and the branching properties of vascular sprouts, and to correlate these events to the phenotype of pericytes.

Such a model is, however, not ideally suited to dissect the role of single ECs. We, therefore, considered generating a mice model to identify and track individual ECs and crossed mice that expressing a nuclear fluorescent protein (histone H2B-EGFP) with the NG2-dsRed strain. Live microscopy monitoring of H2B-EGFP NG2-dsRed mARs allows the detection of individual pericytes and their recruitment to mAR vessels (Figure 2B and Supplementary Movie S3), and also allows visualization of proliferation, migration, and death during the sprouting process with single-cell resolution in both ECs and pericytes.

### 3.3. Pericytes Are Recruited on Newly Formed Sprouting Capillaries and Originate from Proliferative Events within the Aortic Ring

An important step of vascular morphogenesis is pericytes recruitment along the newly formed vessel with the consequent sprout stabilization [21,34,35]. Despite this, the origin of these cells remains an unclear aspect. In the adult organism, mural cells can derive from the bone marrow or myofibroblast and endothelial cell differentiation in response to biochemical or mechanical cues [35,37]. We performed several mAR time-lapse experiments with both transgenic models and never observed any pericyte appearing on mAR sprouts and therefore presumably deriving from ECs differentiation. Therefore, we wondered where these pericytes could originate from.

**Figure 3:**
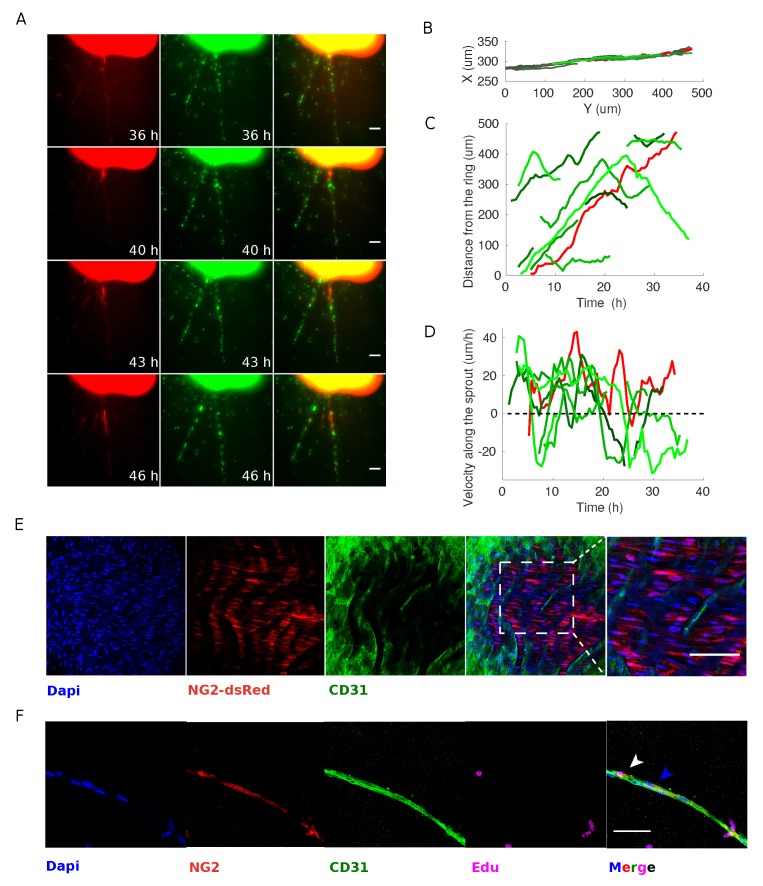
Pericytes recruited on newly formed sprouting capillaries originate from the aortic ring but can proliferate. (**A**) Time-lapse microscopy analysis of H2B-EGFP NG2-dsRed mARs cultured for 5 days then imaged for 72 h. The observed pericyte originates from the edge of the mAR and moves along the ECs during sprouting process (scale bar: 50 μm). (**B**) Cell trajectory analysis shows that endothelial cells and pericytes move along the sprout. This set of trajectories is used for the following two panels. (**C**) The distance of each cell from the ring plotted as a function of time shows that endothelial cells (plotted in different shades of green) move at a rather constant speed and, in some cases, suddenly change direction, as witnessed by the change in slope of some trajectories. (**D**) The velocity of each cell shows that pericytes and endothelial cells do not proceed coherently, but conversely they move independently of each other. In particular cells proceed with bursts (accelerations and decelerations) (**E**) Whole-mount immunofluorescence staining of a NG2-dsRed mA-sheet embedding in a collagen gel and fixed. mA-sheet was stained for the endothelial marker CD31 (green) and DAPI (blue). The rightmost panel shows an enlarged detail. (scale bar: 50 μm). (**F**) mARs were cultured in standard medium supplemented with EdU for 1 week after explant in order to detect whether pericytes and/or ECs coming from the ring originate from a proliferative event or not. Images show a representative experiment. Arrows indicate pericytes Edu-positive (white) or -negative (blue). (Scale bar: 50 μm.)

Taking advantage of the H2B-EGFP NG2-dsRed mouse model we found that the majority of mAR pericytes originate within the aortic ring itself. NG2-positive cells can be detected on the boundary of the ring after typically 5 days of culture and move with respect to ECs during sprouting process (Figure 3A). This strongly suggests that pericytes recruitment might be subsequent to the sprouting of ECs in the angiogenic process, presumably with the aim of stabilizing newly forming vessel.

AS pericytes are only recruited on the sprouts at a later stage, when capillaries are already developed, we wondered if pericytes themselves are dragged by the migrating ECs or whether they are migrating on top of the vBM independently from ECs. To address this question, we performed tracking on single pericytes and ECs on the whole sprouting capillary in H2B-EGFP NG2-dsRed mARs. First, we tracked cells (ECs and pericytes) pertaining to the same sprouting capillary, as shown in Figure 3B,C. From both the time-lapse experiments and the tracks, it is evident that ECs move along the sprout. Therefore, we investigated whether ECs and pericytes would move coherently or whether, instead, we could find uncorrelated displacements and velocities. ECs move typically away from the ring but occasionally can invert the direction of motion and move back towards the ring, as shown in Figure 3C and as previously reported [38]. The overall positioning of the pericytes is also generally consistent with the pericytes following the direction of growth of the capillary, even though accelerations and decelerations can be occasionally observed. This is better depicted in Figure 3D, where it is clear that the velocity of the ECs changes sign, indicating a change of direction, and where it is also evident that pericytes proceed with bursts of migration, i.e., cycles of acceleration and deceleration.

This observation leads to the conclusion that pericytes are not tightly bound to the underlying endothelium, but instead move independently of ECs.

Considering that most of the observed pericytes originate from within mAR, we wondered about their lineage. We hypothesized that NG2-positive cells along the mAR sprout could derive from asymmetric divisions of NG2-negative cells or from pre-existing NG2-positive progenitor cells located on the aorta at the explant time. It has indeed been previously demonstrated that postnatal rat aorta contain an immature subpopulation of mesenchymal NG2-positive cells with pericyte progenitor features [28,39]. We therefore verified the presence and localization of NG2-positive cells within the explanted aortas before sprouting. To this aim, we exploited NG2-dsRed mA-sheets assay which gives better accessibility to the endothelial layer compared to the ring assay. In this assay, mAR are longitudinally cut in order to obtain a square and flat sheet subsequently embedded in collagen gel. The mA-sheets were fixed after gel embedding the same day of aortic explant, and subsequently stained and observed by confocal microscopy. We found a thin layer of NG2-dsRed-positive cells adjacent to the EC layer (Figure 3E). This result demonstrates that the mouse aorta contains a population of NG2-positive cells that may represent an important source of pericytes during sprouting angiogenesis.

To further confirm that mAR NG2-positive pericytes originate within the ring, we evaluated the proliferation rate of this population performing an EdU proliferation assay. LifeAct-EGFP NG2-dsRed mARs were cultured with EdU and observed with confocal microscopy after 7-day incubation. We found that nearly 88% of pericytes were Edu-positive (Figure 3D). This result confirms that pericytes observed in our model derive mainly from proliferative events, likely from pre-existing NG2-positive progenitor cells, and not from purely differentiative events. Interestingly, only 41% of ECs resulted Edu-positive within the elongated sprout confirming that sprouting from mAR is attributable mainly to EC migration rather than proliferation [38]. Notably, we could not detect significant proliferative activity of ECs on the capillary-like structure, as evident by our H2B-GFP NG2-dsRed mARs.

### 3.4. Pericytes Divide on Growing Sprouts and Give Rise to Opposedly Migrating Daughter Cells

In our time-lapse experiments, pericytes migrate along the elongating mAR sprout after exiting the ring. Although pericytes are migrating most of the time, occasional arrests are observed, regardless of the underlying EC flow. Such pauses often coincide with cell division. Moreover, the great majority of analyzed pericytes in the experiments described above, resulted positive for Edu incorporation. For these reasons, we analyzed proliferative events involving pericytes during mAR vessel maturation.

**Figure 4:**
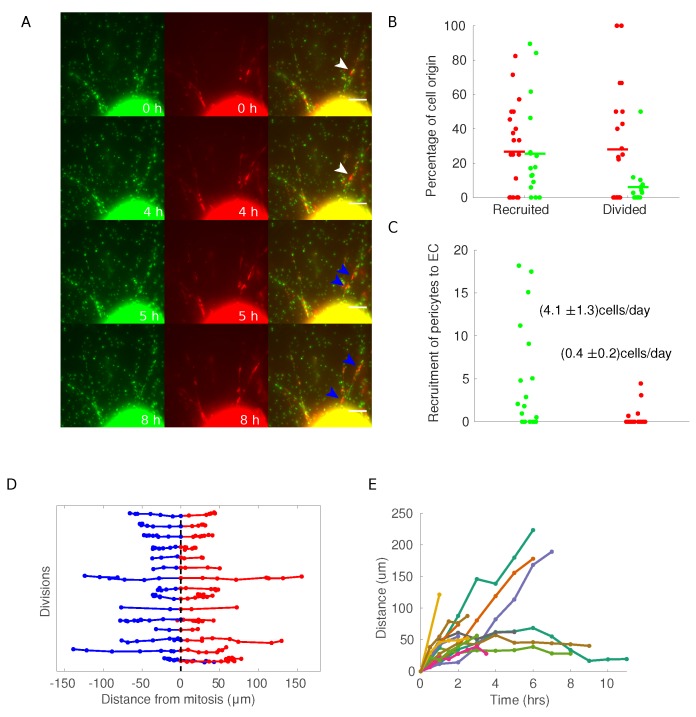
H2B-EGFP NG2-dsRed mAR mouse models to study pericytes dynamics during the sprouting process. (**A**) Time-lapse widefield microscopy analysis of H2B-EGFP NG2-dsRed mARs. White arrow indicate a pericyte that undergoes cell division after 5 h from the beginning of the experiment, generating the two daughter cells marked with blue arrows. (Scale bar: 50 μm.) (**B**) For each cell in a sprout, we verified if it appeared on the sprout coming out of the ring (recruited) or if it divided on the sprout (divided). Each dot represents a single sprout, with green dots for endothelial cells and red dots for pericytes. (**C**) To measure the recruitment rate of cells on the sprouts, we measured the number of cells at the end of each sprouting assay (each dot represents a sprout) and measured the number of cells during the time of the experiment. (**D**) Each line represents a division event of a pericyte. Red and blue trajectories are projections of the trajectories of the daughter cells on the direction of the sprout, where the origin has been defined as the coordinate of the mother cells at the frame preceding mitosis. (**E**) Plot of the distance between daughter cells as a function of time.

By exploiting the H2B-EGFP NG2-dsRed mAR model we could dynamically identify cell division events and follow the daughter cells along the sprout (Figure 2A). Within each sprout, the average number of pericytes with respect to the total number of cells at the end of the assay is (19.7±3.3)% (mean, SEM). Approximately 27% of each component originates from within the ring itself, whereas different fractions (25% for pericytes and 6% for endothelial cells) are generated along the sprout due to cell division (Figure 4B). This strongly support again that the endothelial contribution is mainly driven by endothelial cells coming from the ring, while a significant portion of pericytes is replicated from cells that are already on the sprout, consistently with what has been reported in literature [40,41]. The recruitment of cells from the ring to the sprout proceeds at somewhat different paces for the two cell types. As expected, endothelial cells appear on the sprout on average at the relatively fast rate of nearly 4 cells/day, whereas pericytes exit the ring at a ten-fold lower rate (Figure 4C). Note that these are average rates and could be time-dependent phenomena.

Most NG2-positive cells undergoing mitosis on mARs had quite typical dynamics. Immediately after division, the two daughter cells migrate in opposite directions along the mAR sprout. At longer times, a significant fraction keeps moving in opposite directions along the sprout, i.e., one outward and the other towards the ring. In other cases cells can change direction (Figure 4D). This behavior is evident when considering the distance of the two daughter cells along the sprout, which clearly shows that some cells continue to move away, corresponding to a steady increase in the distance, whereas others display a slowly varying or constant distance at long times (Figure 4D).

## 4. Discussion

We have generated and employed several mouse models that enable following the interactions between ECs and pericytes in a live ex vivo angiogenesis assay. We have shown that endothelial cells forming the new sprouts mainly emerge by collective migration from the core of the pre-existing endothelial layer. The tracking of ECs during sprouting angiogenesis indicates a continuous exchange between “tip” and “stalk” EC, consistent with previous observations in the literature [38,42]. Notably, in our sprouting models, proliferative events involving ECs in the capillary-like structure are very rare, suggesting that ECs proliferation might occur in the inner part of the ring then followed by migration towards the ECM.

It is, however, interesting that ECs and pericytes differ in this regard, as pericytes originate from mitotic events within the mAR but also actively proliferate on the capillary-like structure. Therefore pericyte coverage is guaranteed by both recruitments of progenitors and proliferation of mature pericytes.

It would be interesting to understand the identity of the progenitors of pericytes migrating on the sprouts. This task however poses significant technical problems in our experimental setting. Indeed proliferative events occurring within the mAR are extremely dense of cells, in particular dsRed positive cells, therefore making live-microscopy experiments challenging.

Cell migration of endothelial cells and pericytes is uncorrelated, which has the important direct consequence that contact of pericytes and endothelial cells is indirect through the basal membrane. In this way, pericytes can move on vessels freely and independently on the migration of endothelial cells. This observation has important consequences, as the potential interaction between ECs and pericytes is likely to occur through the exchange of diffusible paracrine signals, rather than direct stable contacts. Given that pericytes and ECs move independently, it is also of great interest to understand what is driving pericyte movement and what are the regulators. It is conceivable that the deposed basement membrane and its remodeling by ECs might play a role and guide the movement of pericytes. This calls for new exciting research directions.

Along the same line, although ECs are guided to form a new sprout by VEGF stimulation, it would be interesting to investigate what regulates the recruitment of pericytes on a newly forming sprout. Indeed, it is necessary for pericytes to have initial proliferative and directional cues, and it is not known whether this occurs through NG2 expressing progenitor cells or other progenitors. Furthermore, it is worth noting that pericytes are recruited on endothelial sprouts that do not obviously have any vascular function yet, supporting the notion that pericytes are needed in early vascular development [1,2,4].

Daughter pericytes proceed in different directions. We have tried to describe what happens to NG2 expression after mitosis. It is conceivable that daughter cells differentiate either into ECs or other cell types. We performed a preliminary analysis exploiting the H2B-EGFP NG2-dsRed mAR model following NG2-positive cells after proliferative events to follow the level of fluorescence intensity of the daughter cells. However, most of our observations are performed with a space resolution that allows monitoring the whole sprout rather than single cells, which would not be compatible with the observation of extremely rare cell division events. Furthermore, we are facing the issue that fluorescence levels start from approximately half the content of the mother cell and either rise to something close to its original level or decrease to zero. Although the rise in fluorescence has the typical time scales of the maturation times, which is reasonably fast and on the order of 70 min [43], the decay has the timescale of proteolytic degradation which can be extremely slow (even of the order of several days) and would therefore require much longer experiments. We, therefore, had to abandon this strategy, even though the biological question is of great relevance.

Due to the inherent multiscale nature of the process studied in both space and time (from cellular to tissue wide level and from dozens of minutes to days), the experimental model we are presenting has several technical limitations together with a number of unique features. First of all it allows dynamic studies of both ECs and pericytes, as the latter have a functional meaning only in relation to the former. It gives the unique opportunity of using a doubly labeled system to study interactions with a third cell type, for example, tumor cells. Due to the low throughput of this assay, it is not suitable to traditional bulk type techniques and requires sophisticated single cell-based methods. Furthermore, sprouts grow in unpredictable directions and spots, making it necessary to constantly monitoring a large region around the explants, with consequent demanding needs in terms of microscopy.

In conclusion, we have presented data that explain the interactions between endothelial cells and pericytes and shed light on the interesting interaction between these two cell types in a biological situation that is extremely close to physiological one.

## Supplementary Materials

The following are available online at https://www.mdpi.com/2073-4409/8/9/1109/s1, Video S1: NG2-dsRed mAR timelapse, Video S2: NG2-dsRed LifeAct EGFP mAR timelapse, Video S3: NG2-dsRed H2B-GFP mARtimelapse.

## Abbreviations

The following abbreviations are used in this manuscript.

VSMC	Vascular Smooth Muscle Cells
EC	Endothelial Cells
vBM	Vascular Basement Membrane
αSMA	alpha-smooth muscle actin
mAR	mouse aortic ring
